# Biomonitoring of Microplastics in Izmir Bay (Eastern Aegean Sea): Abundance, Polymer Characterization, and Ecological Risk Assessment in Saddled Seabream, *Oblada melanurus* (Linnaeus, 1758)

**DOI:** 10.3390/ani16162524

**Published:** 2026-08-13

**Authors:** Rahşan Evren Mazlum, Arzu Aydın Uncumusaoğlu, Bahar Bayhan, Tanju Mutlu, Kaan Karaoğlu, Ali Kara, Burcu Taylan

**Affiliations:** 1 Department of Fishing and Processing Technology, Faculty of Fisheries, Recep Tayyip Erdoğan University, 53100 Rize, Türkiye; 2 Department of Environmental Engineering, Faculty of Engineering, Giresun University, 28200 Giresun, Türkiye; 3 Department of Hydrobiology, Faculty of Fisheries, Ege University, 35100 Izmir, Türkiye; 4 Vocational School of Technical Sciences, Recep Tayyip Erdogan University, 53100 Rize, Türkiye; 5 Climate Change and Environmental Problems Application and Research Centre, Recep Tayyip Erdoğan University, 53100 Rize, Türkiye; 6 Department of Fishing and Processing Technology, Faculty of Fisheries, Ege University, 35100 Izmir, Türkiye

**Keywords:** microplastic pollution, *Oblada melanurus*, Polymer Hazard Index (PHI), temporal dynamics, Aegean Sea

## Abstract

In this study, we aimed to investigate the occurrence, characteristics and temporal dynamics of microplastics (MPs) in the gastrointestinal tracts of saddled seabream (*Oblada melanurus*) from Izmir Bay (Eastern Aegean Sea). Sampling was carried out monthly during a one-year period (March 2021–February 2022) and MPs were identified by stereomicroscopy and ATR-FTIR spectroscopy. Microplastics were identified in 115 of the 180 examined individuals, representing an overall frequency of occurrence (FO) of 63.89%. A total of 298 microplastic items were recovered, corresponding to a mean abundance of 1.66 ± 0.35 items individual^−1^ across all examined fish (N = 180) and a mean intensity of 2.59 ± 0.41 items/individual among positive individuals (N = 115). The highest occurrence (100%) was observed during December. Fibers were the most abundant morphotype (58%) followed by fragments (39%). Black (56%) and blue (20%) particles were the most abundant colors. Size distribution was 105–4678 µm with mean length of 1370.08 ± 1122.74 µm. The most frequently identified polymers were polyethylene terephthalate (PET, 29%), polyethylene (PE, 19%) and polypropylene (PP, 18%). We did not find any significant relationships between MP abundance and fish size, weight or sex (*p* > 0.05). The study area was classified into high-risk category (Category IV) based on the value of Polymer Hazard Index (PHI) (129.52). The results show temporal variability of MP ingestion, likely influenced by seasonal conditions and anthropogenic activities.

## Simple Summary

Plastic pollution poses a growing threat to marine ecosystems because many marine animals can ingest small plastic particles. These particles can affect animal health and indicate the level of pollution in coastal waters. This study investigated the presence of small plastic particles in the digestive tracts of saddled seabream, an important coastal fish species in Izmir Bay, Türkiye, over a one-year period. Almost two-thirds of the examined fish had ingested plastic particles, with the highest occurrence recorded in winter. The majority of the particles were fibers, followed by small fragments. Black and blue particles were the most common colors. Several common plastic materials used in textiles, packaging, and consumer products were identified. Plastic ingestion did not appear to be related to the size, weight, or sex of the fish. The results also indicated that the study area has a relatively high level of plastic pollution. These findings improve our understanding of how plastic pollution changes throughout the year and highlight the need for effective measures to reduce plastic waste entering the marine environment, helping to protect marine life and coastal ecosystems.

## 1. Introduction

Pollution of seas and oceans with anthropogenic debris has continued to attract increasing attention from scientists over the past century as a global environmental issue [1]. Although the first article on plastic pollution in the oceans was published in 1972 [2], the term *microplastic* (MP) was first used in 2004, and this study accelerated research conducted on MPs [3,4]. According to 2023 data, the total global plastic production was 413.8 million tons, and 8–10 million tons of plastic enter the oceans each year [1,5]. Due to their versatile properties and low cost, plastics have become a globally widespread material [6,7]. Because plastics degrade very slowly under natural environmental conditions, they tend to persist in the environment, gradually fragmenting into microplastics [1]. As plastics fragment into smaller particles, the abundance of MPs in marine environments increases and consequently, this phenomenon enhances the potential impact on aquatic organisms [8]. Recent estimations suggest that 50–75 trillion polymer particles are present in water [1]. Plastic materials have the potential to cause adverse effects globally as a result of their entry into natural and aquatic ecosystems [9,10].

The term microplastics (MPs), which is used by most authors, generally refers to plastic particles that have a diameter of less than 5 mm [11,12,13]. Others argue that, in order to refer only to particles produced when various plastic-based products are exfoliated and decomposed in ecosystems, the word microplastics should be modified to *“items < 1 mm”* [10,14,15]. MPs are present everywhere in marine environments and coastal areas due to ocean waves and currents. The aquatic ecosystem contains MP particles of different sizes, densities, chemical compositions, and shapes [16,17,18].

Due to inefficient waste management, the prevalence of single-use plastics, and industrial waste, marine environments, surface waters, sediments, and biota are negatively affected by this situation [1,7,19]. MPs are found in a wide variety of aquatic organisms, including fish, bivalves, mammals, crabs, sea snakes, and seabirds [8,20]. A large proportion of organisms living in aquatic ecosystems mistakenly ingest plastic waste released into the water because they misidentify these materials as a food source [18,21,22]. Almost all aquatic organisms ingest MPs, and there are significant differences in ingestion volumes among these species [23,24]. The total amount of consumed MPs varies depending on the organism and location, and even within the same region, significant differences can be observed [24].

The ingestion of MPs can have several detrimental effects on aquatic organisms. Feeding retardation has been reported as one of the physiological effects of MP ingestion, and the ingestion of a greater number of particles may represent a higher risk to the consuming species [25,26]. In addition, plastics contain hazardous additives, such as plasticizers, antioxidants, flame retardants, and pigments, which may cause lethal effects and bioaccumulation [27]. Furthermore, MPs can act as vectors for environmental contaminants, including polycyclic aromatic hydrocarbons (PAHs), polychlorinated biphenyls (PCBs), DDT, and organohalogen pesticides, which can accumulate at high concentrations on their surfaces and facilitate the transfer of these pollutants into aquatic organisms [28].

MPs are considered resistant to biotic degradation [23,24]. The ingestion of MPs by key species at lower trophic levels, which are negatively affected, and their subsequent consumption by species at higher trophic levels cause problems in energy and nutrient transfer within these organisms. Consequently, studies have shown that ingested MPs accumulate in aquatic organisms and can lead to stress responses and oxidative stress, which affect the animal’s growth, metabolism, behavior, and reproduction [3,29,30,31,32,33,34].

Izmir Bay is a semi-enclosed natural bay located in the Aegean Sea (38°41′–38°21′ N, 26°30′–27°08′ E) [35,36,37]. With a surface area of over 500 km^2^ and a water volume of 11.5 billion m^3^, Izmir Bay is the largest natural bay in the Mediterranean [36,37]. Hosting a rich marine ecosystem and many commercially valuable fish species, Izmir Bay is an important fishing area for both the Aegean Sea and Turkey [38]. According to TURKSTAT (2024), the catch amount of *O. melanurus* was 127.9 tons in 2014, whereas in 2024 this value decreased by approximately 60% to 77.2 tons [39]. Numerous published studies indicate that this decline in catch is closely related to overfishing, pollution, global warming, and invasive species [40,41,42]. The Gediz River, the second largest river of the Eastern Aegean coast, flows into the northern part of the bay. Along its coast, there is dense settlement as well as numerous manufacturing, food, and chemical industries [36]. Consequently, the bay is affected by pollutants from coastal areas [37]. Intense economic activities and negative effects of plastic pollution on marine and human life are frequently observed in regions with high plastic accumulation [43,44].

The saddled seabream, *Oblada melanurus* (Linnaeus, 1758), is a widespread benthopelagic species distributed throughout the Mediterranean Sea and the Eastern Atlantic, where it typically inhabits coastal waters at depths of up to 30 m [45,46]. It is an omnivorous fish that feeds mainly on small invertebrates and occupies an intermediate trophic level (3.38) [45]. These ecological characteristics make the species relevant for investigating dietary exposure to microplastics in coastal marine environments. Despite its wide distribution and commercial importance, biological and ecological studies on *O. melanurus* remain limited. Previous research has focused primarily on its age, growth, and reproductive biology [47,48,49,50,51,52,53].

The study conducted by Bayhan and Uncumusaoğlu (2024) provided an essential preliminary assessment of ecological risks associated with MP accumulation in several commercial fish species within Izmir Bay [38]. Their research encompassed 366 individuals across 13 species, reporting an average MP abundance of 2.21 ± 0.47 items per individual. However, while that study offered a valuable broad-spectrum overview, the limited sample size per species and the multi-taxa approach did not allow for a high-resolution, species-specific evaluation or the tracking of temporal (monthly) variations. Consequently, the present study focuses exclusively on *Oblada melanurus* to fill these critical data gaps. By employing a more robust sampling size and monthly monitoring, this research aims to comprehensively determine the abundance, morphological characteristics, and chemical composition (via μ-FTIR) of MPs, while providing a detailed ecological risk assessment specific to this bio-indicator species. We hypothesized that microplastic ingestion would exhibit monthly variation, that polymer composition would reflect the dominant sources of plastic pollution in Izmir Bay, and that microplastic abundance might be associated with fish biometric characteristics.

## 2. Materials and Methods

### 2.1. Study Area and Sample Collection

**Figure 1:**
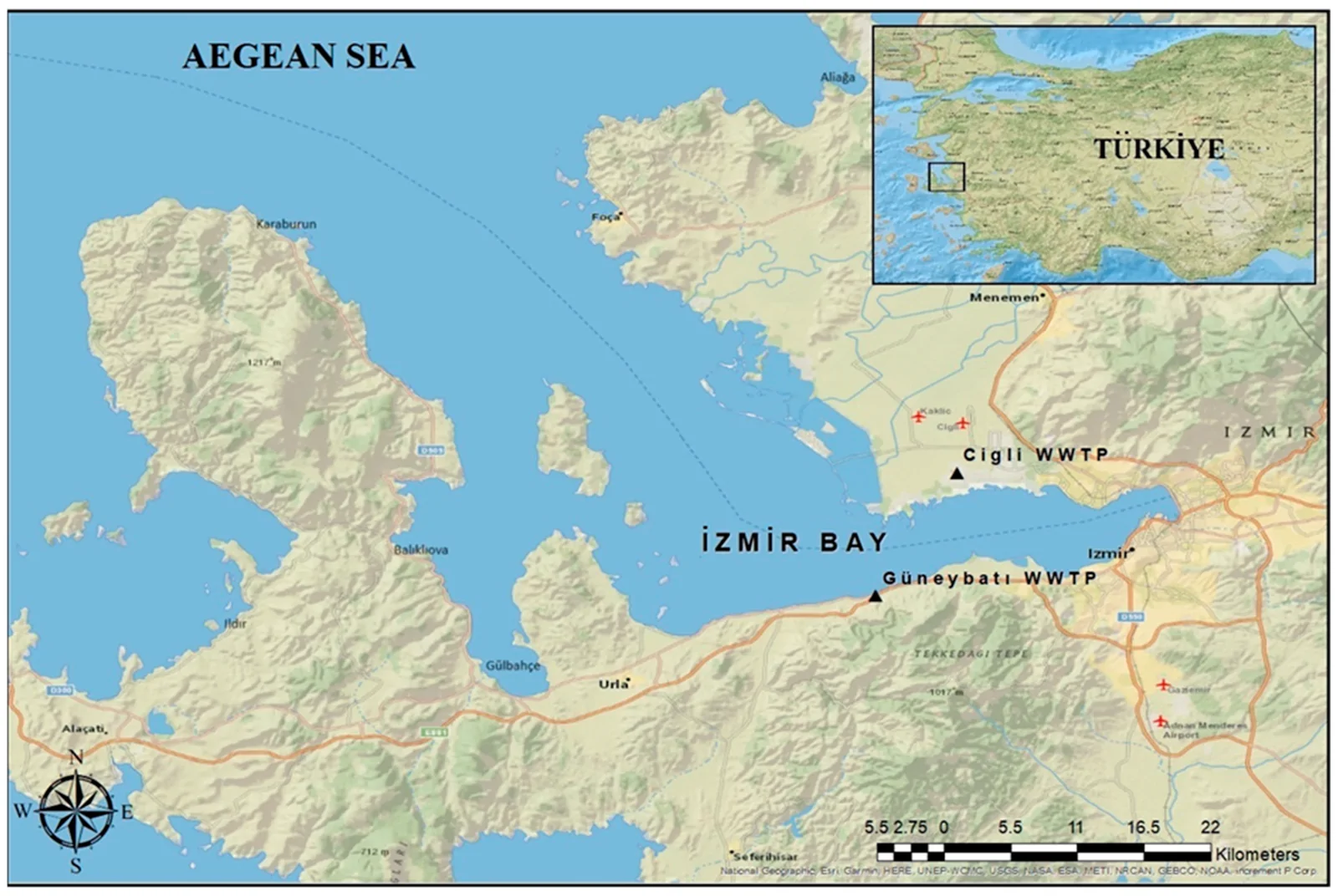
Map of the study area and sampling stations for *Oblada melanurus* in Izmir Bay (Eastern Aegean Sea, Türkiye). (The map was generated using ArcGIS 10.8.1).

Izmir Bay, located on the eastern coast of the Aegean Sea, is a large natural embayment in the Aegean Sea (Figure 1). With a coastline stretching roughly 629 km, Izmir is one of the most significant fishing hubs in Türkiye, hosting diverse fish families such as Clupeidae, Sparidae, Carangidae, and Scombridae. The saddled seabream (*Oblada melanurus*) is a key coastal species widely harvested in the bay for both its ecological role and economic value. This species typically inhabits rocky areas and *Posidonia* beds, which are highly susceptible to anthropogenic pressures including urban runoff, maritime traffic, tourism, and intensive fishing [54].

Izmir is the third-largest metropolis in Türkiye, characterized by dense industrial, agricultural, and residential activities. The Gediz, Küçük Menderes, and Bakırçay rivers are the primary conduits transporting domestic and industrial microplastics (MPs) into the bay. Despite the operation of a large-scale wastewater treatment plant since 2000, the bay remains an at-risk ecosystem for MP pollution.

In this study, a total of 180 *O. melanurus* specimens were collected monthly (*n* = 15 per month) between March 2021 and February 2022. To avoid spatial confounding effects and ensure consistent monitoring, sampling was carried out across fixed sampling stations in the inner and outer areas of Izmir Bay (Figure 1). At each monthly sampling event (conducted between the 15th and 20th day of each month), an equal number of specimens (5 individuals per station) were collected using standardized traditional monofilament trammel nets (inner mesh size: 32–36 mm; outer mesh size: 160 mm; net height: 1.5–2.0 m). Fishing operations were performed by local artisanal fishers at depths between 10 and 25 m, corresponding to the typical benthopelagic habitat of *O. melanurus*. Sea surface temperature, salinity, and precise GPS coordinates for each deployment were recorded. Immediate wrapping in aluminum foil and storage at −20 °C ensured sample integrity prior to laboratory dissection.

For each specimen, total length (TL) was measured to the nearest millimeter using a measuring board. Body and gastrointestinal (GI) tract weights were recorded using a precision balance (0.01 g). Sex was determined macroscopically, and descriptive statistics are summarized in Table 1. Fresh samples were immediately wrapped in aluminum foil and stored at −20 °C until GI tract dissection and polymer analysis [55].

### 2.2. Sample Treatment and MP Extraction

A total of 180 *Oblada melanurus* specimens (*n* = 15 per month) were processed. Dissections were performed on a stainless-steel tray using sterile surgical tools. Gastrointestinal (GI) tracts were carefully removed, weighed, and transferred to individual 500 mL glass beakers.

To digest organic matter, a modified Fenton’s oxidation protocol was applied following standardized NOAA guidelines [56] and optimized biota procedures [57]. Briefly, 20 mL of a freshly prepared 0.05 M Fe(II) solution (prepared using FeSO_4_·7H_2_O and acidified to pH ~3.0 with concentrated H_2_SO_4_) was added to each sample beaker. Subsequently, 20 mL of 30% H_2_O_2_ (Sigma-Aldrich, Gillingham, UK) was added incrementally in 5 mL aliquots to control the exothermic reaction, avoid violent foaming, and prevent thermal degradation of low-melting-point polymers [57]. Beakers were loosely covered with aluminum foil and incubated in a shaking incubator at 50 °C and 80 rpm for 24 h. If organic tissue dissolution was incomplete, additional 5 mL aliquots of 30% H_2_O_2_ were added at room temperature until complete digestion was achieved.

Following digestion, density separation was performed using a filtered, saturated NaCl solution (density ~ 1.20 g cm^−3^) [56]. Samples were transferred to glass Imhoff cones, diluted with saturated NaCl, and allowed to settle for 10 days to maximize particle flotation and settling. The supernatant was vacuum-filtered through 1.2 μm glass fiber filters (Whatman GF/C, GE Healthcare, Buckinghamshire, UK). Filters were dried in an oven at 40 °C for 48 h prior to microscopic analysis.

Saturated NaCl is widely recognized for its cost-effectiveness and environmental safety; however, its density threshold (1.20 g cm^−3^) may under-recover high-density polymers such as polyethylene terephthalate (PET, ρ ≈ 1.38 g cm^−3^) compared to high-density flotation media like ZnCl_2_ or NaI [57,58]. Although high-aspect-ratio PET fibers and fragments were recovered in this study—facilitated by prolonged settling times and hydrodynamic lift—the reported abundance values represent a conservative baseline estimate for dense synthetic polymers.

### 2.3. Observation and Identification of MPs

Dried filters were examined under a stereo microscope (Nikon, Tokyo, Japan). Particles were categorized by size (measured via integrated digital camera), color (red, green, blue, black, yellow, and transparent), and morphology (film, fragment, fiber, foam, and pellet) [38]. A hot needle test was applied to suspicious particles to confirm their plastic nature [59].

### 2.4. FTIR Spectroscopy

Chemical identification of potential microplastics was performed using a Bruker VERTEX 70 FTIR spectrometer equipped with a Diamond Attenuated Total Reflectance (ATR) accessory (Bruker Optics GmbH & Co. KG, Ettlingen, Germany). A representative 20% subsample of visually isolated particles (including 100% of particles larger than 500 μm) was individually transferred onto the ATR crystal. Spectra were acquired in transmittance/absorbance mode across the wavenumber range of 4000–400 cm^−1^ at a resolution of 4 cm^−1^ with 32 co-added scans.

Spectral matching was performed using OPUS software (version 8.7) against established reference databases, including the Bruker OPUS Polymer Library and the Kramer Synthetic Fiber Library. A strict numerical match threshold of ≥80% quality index was applied for positive polymer verification. Spectra exhibiting hit quality scores between 70% and 79% underwent manual baseline correction and atmospheric compensation; particles remaining below the 80% threshold were designated as unidentified and excluded from total microplastic counts.

Inorganic mineral phases (e.g., calcite, CaCO_3_) were identified but excluded from microplastic abundance calculations. Regenerated cellulosic fibers (rayon and lyocell), while derived from natural cellulose, were included due to their anthropogenic processing and persistent environmental release; however, they are distinctly reported as Anthropogenic Cellulosic Fibers (ACFs) in accordance with standardized reporting frameworks [60].

### 2.5. Quality Assurance/Quality Control (QA/QC)

Strict Quality Assurance and Quality Control (QA/QC) protocols were implemented throughout the analytical process to prevent background and procedural microplastic contamination. All laboratory procedures were conducted in a controlled environment. Personnel wore 100% cotton laboratory coats and powder-free nitrile gloves. To minimize external contamination, only intact fish specimens were processed; their gastrointestinal (GI) tracts were thoroughly rinsed with deionized water prior to dissection to ensure that the analyzed particles reflected ingested plastics rather than surface adherence.

Non-plastic equipment was used exclusively; all glassware and stainless-steel tools were rinsed three times with 0.45 μm filtered distilled water and kept covered with clean aluminum foil when not in use. Chemical reagents, including the saturated NaCl solution and 30% H_2_O_2_, were pre-filtered through 0.45 μm membrane filters prior to application.

To monitor potential airborne and reagent contamination, three procedural blanks (*n* = 3) containing all chemical reagents without tissue matrix were processed in parallel and exposed to laboratory air during the analytical steps. Microscopic and ATR-FTIR inspection of these procedural blanks revealed no microplastic contamination (0.0 particles per blank), confirming the efficacy of the implemented anti-contamination measures.

While these measures effectively controlled background contamination, we acknowledge that the use of three procedural blanks across the sampling period and the lack of batch-specific blank corrections and positive spike-recovery trials constitute a methodological limitation of this study. Consequently, the reported microplastic abundances represent unadjusted baseline values.

### 2.6. Statistical Analysis

Statistical evaluations of the data were performed using IBM SPSS Statistics (Version 26.0, IBM Corp., Armonk, NY, USA) and PAST software (Version 4.13). Prior to statistical testing, the normality of the data distribution was assessed using the Shapiro–Wilk test, and the homogeneity of variances was evaluated using Levene’s test. Because microplastic count data were discrete, included zero values, and violated normality and homoscedasticity assumptions (*p* < 0.05), non-parametric statistical procedures were applied.

To evaluate significant differences in microplastic (MP) abundance across the monthly sampling periods, the non-parametric Kruskal–Wallis H test was performed. When significant spatial or temporal differences were detected, Dunn’s post hoc test with Bonferroni adjustment was used for pairwise comparisons. For all Kruskal–Wallis tests, the test statistic (*H*), degrees of freedom (*df*), exact adjusted *p*-values, and effect size (Ƞ^2^_H_) were reported.

The relationships between biological parameters (total length, body weight, and gastrointestinal weight) and the number of ingested microplastics were evaluated using Spearman’s rank correlation analysis (*p*). To identify patterns and similarities among microplastic characteristics (polymer types, colors, and shapes) across sampling months, multivariate statistical techniques were applied. Principal Component Analysis (PCA) was used to reduce dataset dimensionality and visualize major contributing factors, while Hierarchical Cluster Analysis (HCA) was conducted using Ward’s method with Euclidean distance to group parameters based on similarity.

For all statistical tests, the significance level was set at *p* < 0.05. Exact *p*-values are reported, and values less than 0.001 are expressed as *p* < 0.001 rather than *p* = 0.000.

### 2.7. Potential Risk Assessment of Microplastics

The potential ecological and chemical risk of the identified microplastics was evaluated using the Polymer Hazard Index (*PHI*), following the established methodologies of Lithner et al. (2011), Ranjani et al. (2021), Aydin Uncumusaoglu (2024), and Kilic and Uncumusaoglu (2024) [61,62,63,64]. This index assesses chemical risk based on the chemical toxicity of constituent monomers and the associated hazard statements of each polymer type. The overall and monthly *PHI* values for the microplastics detected in *O. melanurus* were calculated using the following equation:

(1) PHI=∑(Pn∗Sn)

where *Pn* is the relative percentage abundance of each polymer type relative to the total synthetic microplastic pool, and *Sn* is the hazard score assigned to that polymer based on monomer toxicity [61]. To ensure reproducibility and consistency with established polymer risk assessment frameworks, inorganic mineral phases (calcite, CaCO_3_) were excluded from the denominator of microplastic abundance calculations and were not included in Polymer Hazard Index (*PHI*) calculations. Anthropogenic Cellulosic Fibers (rayon and lyocell) were excluded from the *PHI* hazard scoring, as standardized monomer toxicity hazard scores (*Sn*) in established models are formulated strictly for synthetic polymers [61]. However, ACFs are reported alongside the dataset as anthropogenic fibers for full transparency. A comprehensive list of every identified material, its relative abundance (*Pn*), assigned hazard score (*Sn*), the source of the score, and its individual contribution (*Pn* × *Sn*) to the overall *PHI* score is provided in Table 2. Monthly composition and chemical risk variations were visualized using a row-normalized Polymer-by-Month Distribution Heatmap, generated using PAST software (version 4.13). The overall and monthly calculated scores were evaluated against the risk categories defined by Ranjani et al. (2021) [Category I (<1, Minor), Category II (1–10, Medium), Category III (10–100, High), Category IV (100–1000, Danger), and Category V (>1000, Extreme Danger)] to evaluate the temporal severity of the chemical threat [62].

## 3. Results

### 3.1. Abundance and Occurrence of Microplastics

A total of 180 *O. melanurus* individuals were examined to evaluate microplastic (MP) contamination in their gastrointestinal (GI) tracts. MPs were identified in 118 individuals, representing an overall occurrence rate of 63.89%, while 65 individuals (36.11%) showed no detectable MP ingestion. From the positive samples, a total of 298 MP particles were isolated.

**Figure 2:**
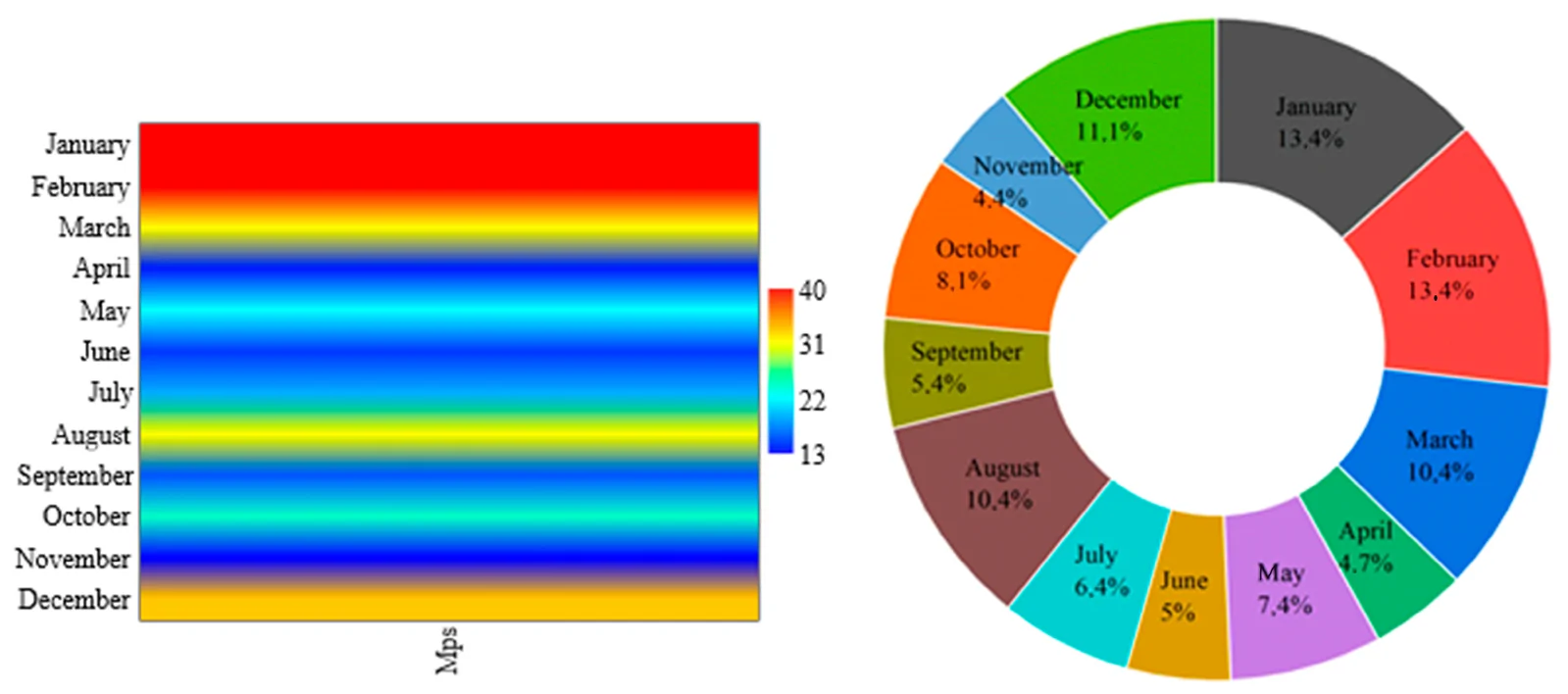
The monthly variation in the abundance of MPs in the GI tract of *Oblada melanurus*.

The monthly variation in MP abundance showed statistically significant differences (Kruskal–Wallis, *p* < 0.05), as illustrated in Figure 2. The highest MP densities were recorded in January and February, both reaching 2.67 items individual^−1^. Notably, in December, MPs were detected in 100% of the sampled individuals, although the numerical density was lower than in the peak months. Conversely, the minimum MP abundance was observed in November (0.87 items individual^−1^). The overall mean MP abundance for the entire study period was calculated as 2.59 ± 0.41 items individual^−1^.

Statistical analysis indicated that microplastic (MP) ingestion was independent of the biological and morphological characteristics of the fish. No significant relationship was found between MP abundance and the sex of the specimens (*p*> 0.05). Similarly, correlation analyses showed that MP uptake showed no statistically meaningful relationship ry with total length (TL), body weight (W), or gastrointestinal tract weight (GW) (Kruskal–Wallis and Spearman Rank Correlation, *p* > 0.05 for all parameters). These findings suggest that MP exposure for *O. melanurus* in Izmir Bay is primarily driven by seasonal environmental dynamics and opportunistic feeding behavior rather than specific biological traits or growth stages of the fish.

### 3.2. Polymer Composition and Temporal Distribution

**Figure 3:**
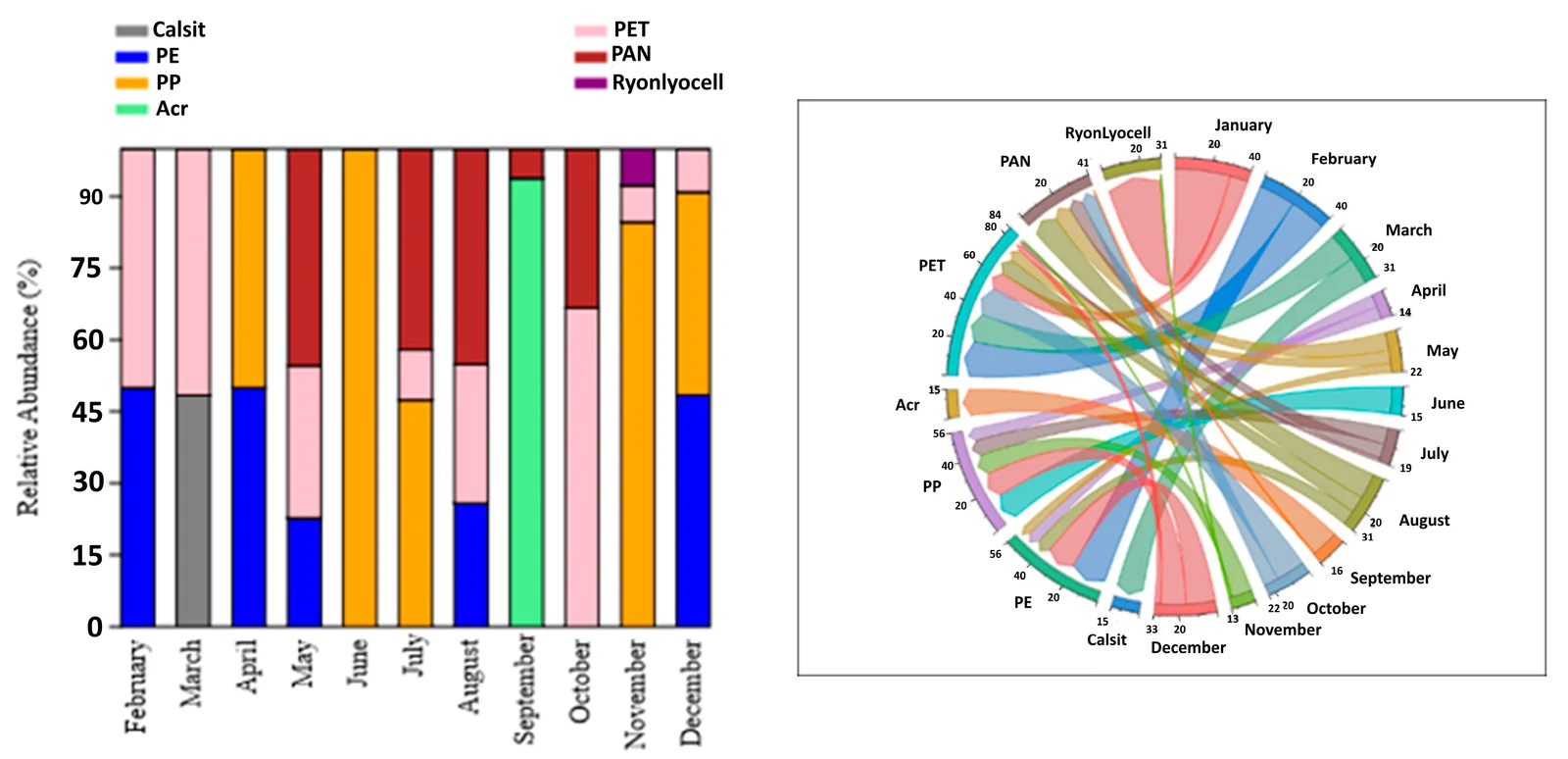
Polymer compositions of microplastics in the gastrointestinal tract (GIT) of *Oblada melanurus* sampled from Izmir Bay.

A total of seven distinct polymer types were identified via ATR-FTIR analysis. The most prevalent polymer was polyethylene terephthalate (PET), accounting for 29% of the total, followed by polyethylene (PE, 19%), polypropylene (PP, 18%), and polyacrylonitrile (PAN, 14%). Other identified polymers included rayon/lyocell (10%), calcite (5%), and acrylic (4%) (Figure 3). The temporal distribution of these polymers showed high variability. PET was identified across seven different months (Jan., Feb., Mar., May, Jun., Aug., and Nov.), while PAN was observed in five months, primarily during the summer and early autumn. Certain polymers were found exclusively in specific periods: acrylic in September, calcite in March, and rayon/lyocell in January. Statistical analysis revealed that the abundance of all identified polymer types varied statistically across the sampling months (Kruskal–Wallis, *p* < 0.001) for all types.

**Figure 4:**
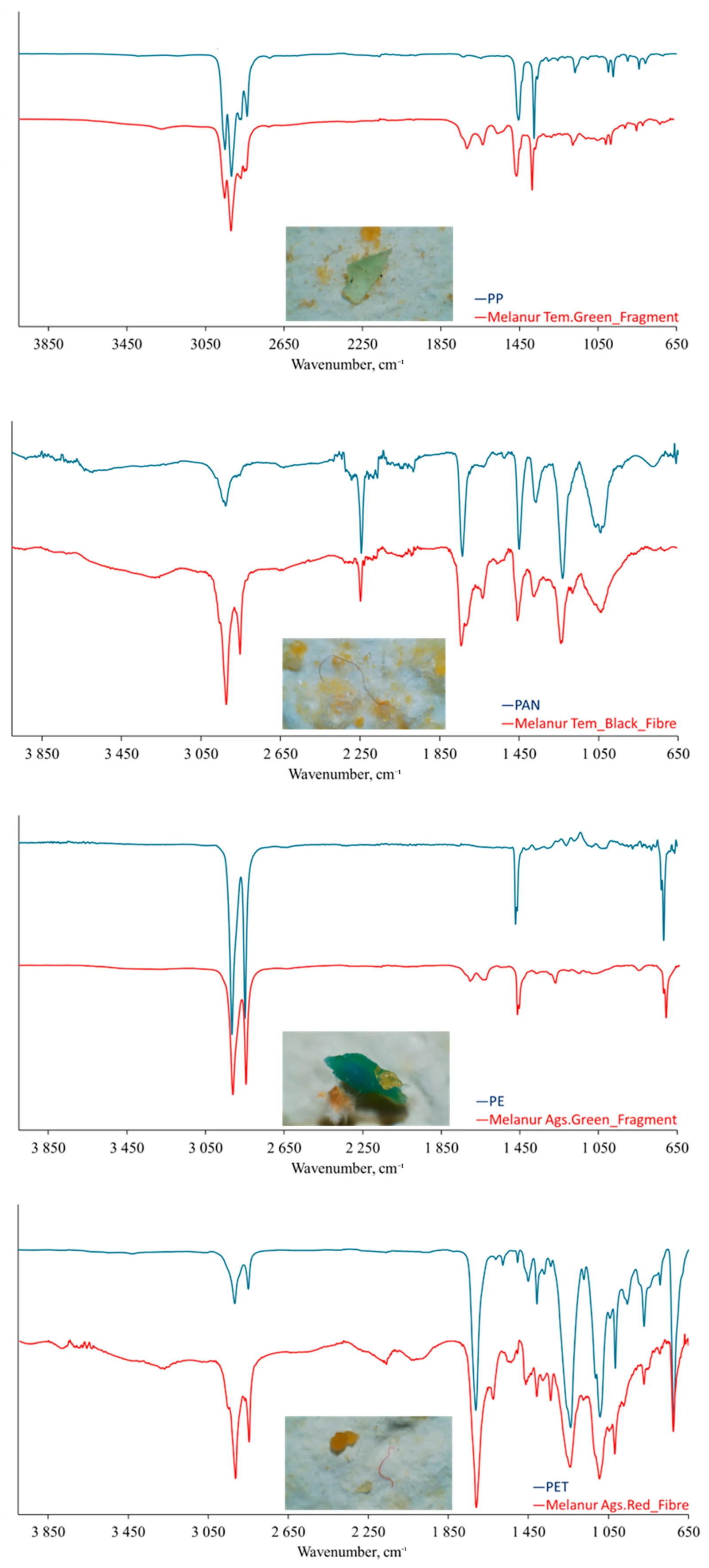
Representative μ-FTIR spectra of microplastics identified in *Oblada melanurus.* Comparison between the reference spectra (blue lines) and the sample spectra (red lines) obtained from microplastics isolated from *O. melanurus*.

The chemical composition of the extracted microplastics was verified using ATR-FTIR spectroscopy, with representative spectra shown in Figure 4. The analysis confirmed a high degree of similarity between the samples and reference polymers. Specifically, the spectra of black fibers and red fragments showed a clear match with polyacrylonitrile (PAN) and polyethylene terephthalate (PET), respectively.

### 3.3. Physical Characteristics of Microplastics: Morphotypes, Colors, and Size Distribution

**Figure 5:**
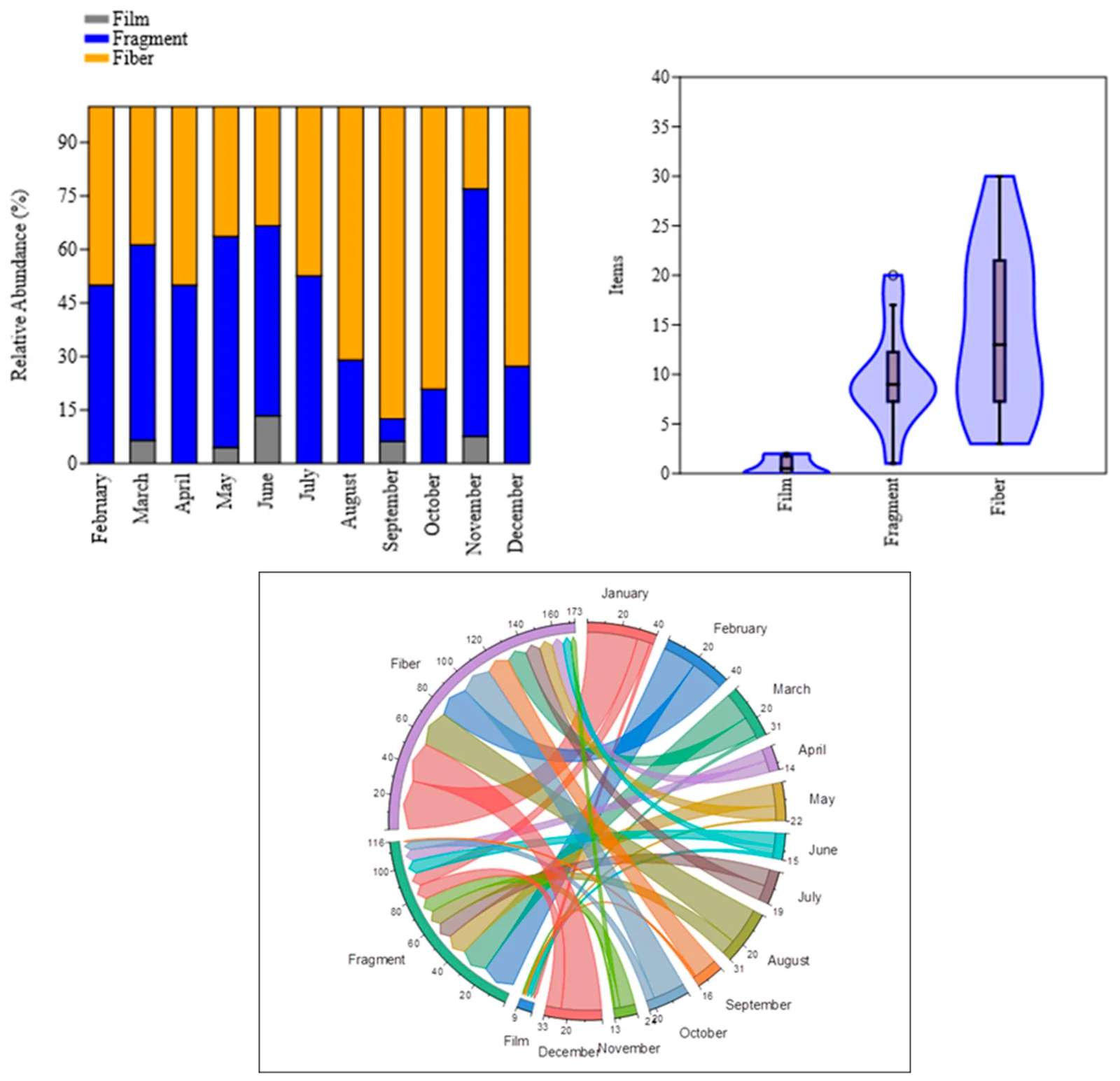
Characterization of microplastics in the gastrointestinal tract (GIT) of *Oblada melanurus* sampled from Izmir Bay.

The morphological analysis of the 298 isolated microplastics (MPs) revealed distinct patterns in shape and color across the sampling period. Fibers were the most dominant morphological form, accounting for 58% of the total MPs, followed by fragments (39%) and films (3%) as shown in Figure 5. Statistically, significant monthly variations were observed for both fibers (*p* < 0.001) and fragments (*p* = 0.001) (Kruskal–Wallis, *p* < 0.05). While fibers and fragments were present in almost every sampling month, films were detected only in January, March, June, and December. From March to November (excluding some months), fragments frequently emerged as the predominant form, reflecting a shift in the degradation state of plastics in the bay.

**Figure 6:**
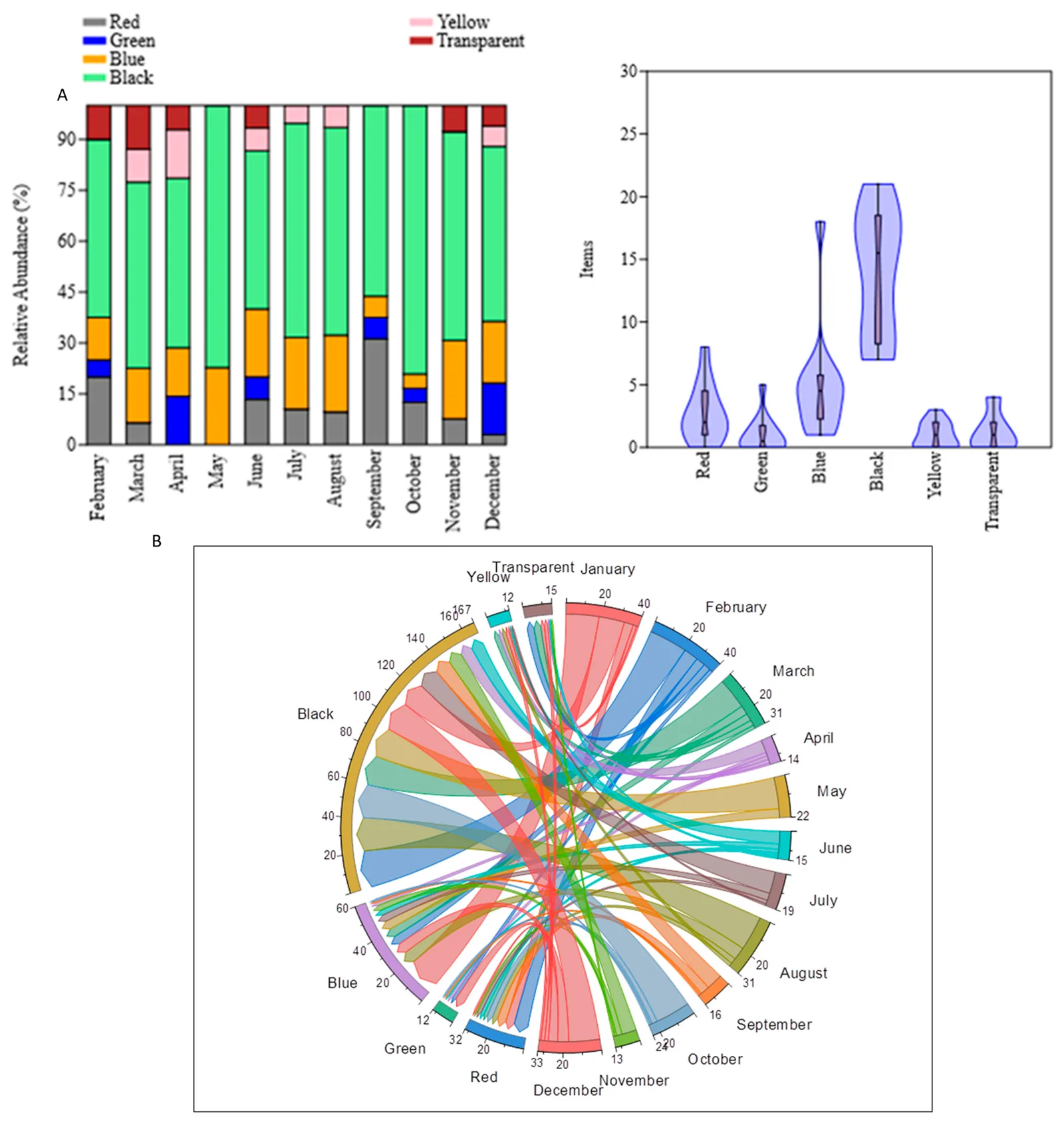
Color distribution and temporal variation in microplastics in *Oblada melanurus*. Analysis of microplastic colors identified in the gastrointestinal tracts: (**A**) Overall percentage distribution of the six identified colors, and (**B**) monthly variation in color frequencies throughout the sampling period. Black and blue were the most persistent and dominant colors across all months. Statistical significance (*p* < 0.05) was observed only for the monthly distribution of blue particles.

The color distribution analysis, illustrated in Figure 6, revealed the presence of six distinct color categories among the isolated microplastics. Black (56%) and blue (20%) were identified as the most prevalent colors, collectively accounting for more than three-quarters of the total particles. These were followed by red (11%), transparent (5%), green (4%), and yellow (4%) (Figure 6). Throughout the entire study period, black remained the consistently dominant color across all sampling months. Interestingly, statistical analysis indicated that except for blue-colored particles (*p* = 0.003), there were no significant monthly variations in the presence of other color groups (*p* > 0.05). Additionally, rare white-colored particles were exclusively detected during February and August, indicating a highly specific temporal occurrence for certain color types. As illustrated in Figure 6, black and blue particles constituted the majority of the recovered plastics. The temporal analysis (Figure 6) further demonstrates the persistence of these colors, with black being present in every sampling month regardless of seasonal changes.

**Figure 7:**
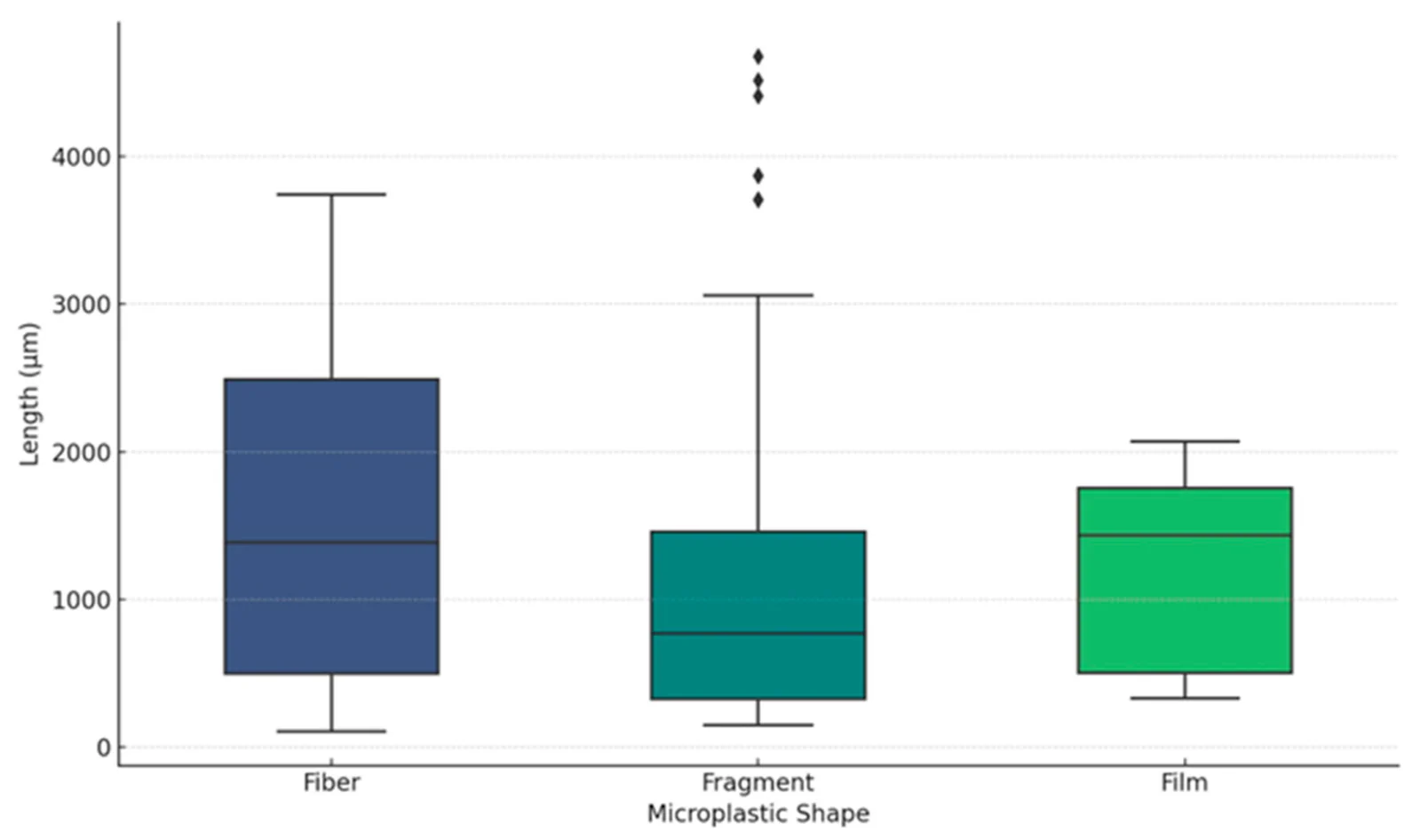
Size distribution of microplastics isolated from the GIT of *Oblada melanurus*.

The identified MPs ranged in size from 105 to 4678 µm, with an overall mean length of 1370.08 ± 1122.74 µm (Figure 7). When categorized by shape, the average lengths were 1550 ± 1126 for fibers, 1160 ± 1124 for fragments, and 1254 ± 656 for films. Statistical analysis indicated a significant difference in MP length among the different morphological types (*p* < 0.05), suggesting that the physical degradation and transport dynamics vary depending on the shape of the plastic debris.

### 3.4. Potential Risk Assessment of Microplastics (PHI)

**Figure 8:**
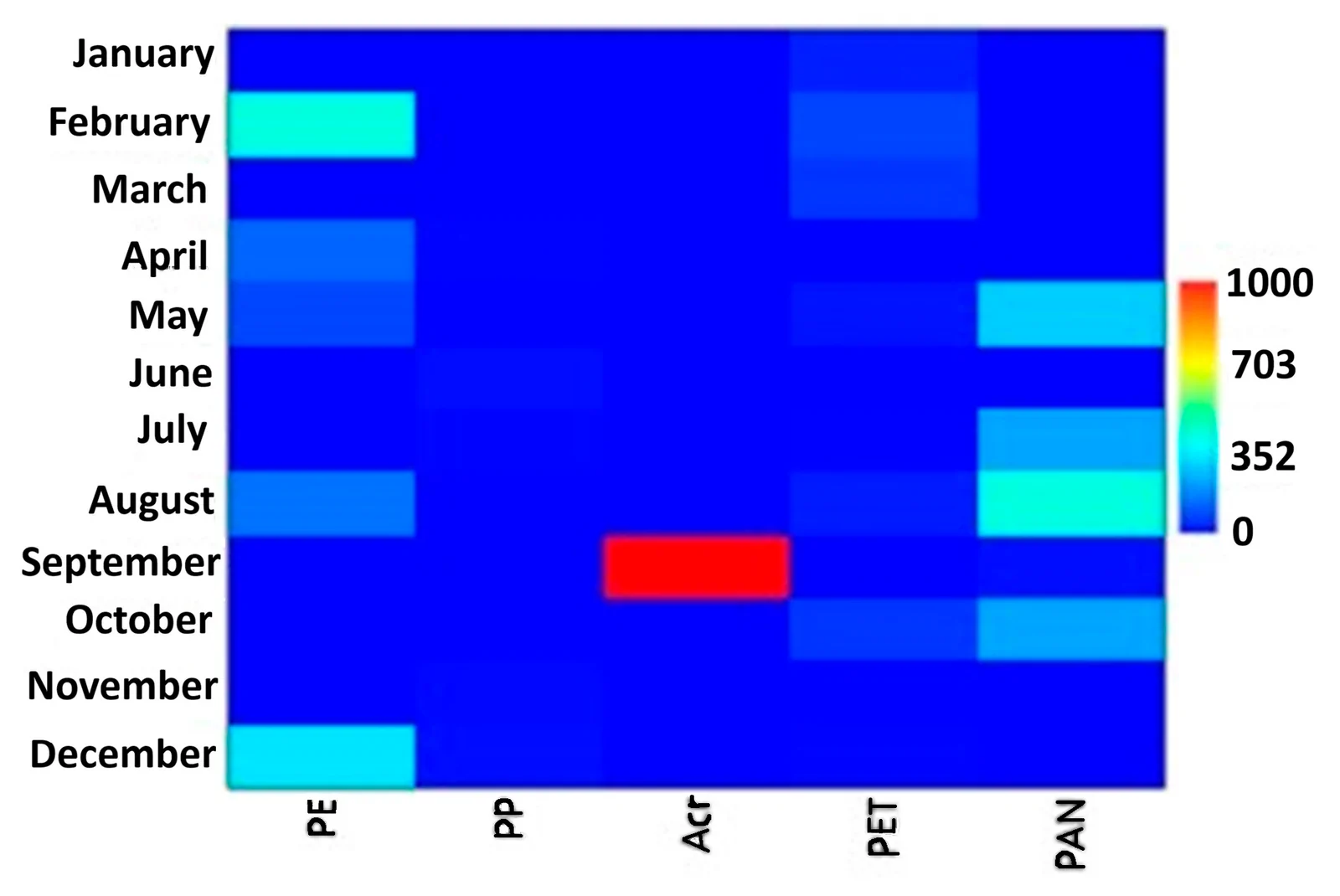
Heatmap distribution of monthly Polymer Hazard Index (PHI) and polymer composition across sampling months.

To evaluate the chemical threat posed by these polymers, the Polymer Hazard Index (PHI) was calculated based on the chemical toxicity of the monomers. The overall PHI for *O. melanurus* in Izmir Bay was determined to be 129.52. According to the ecological risk classification, this value places the study area in Hazard Category IV (Danger). The presence of relatively high-hazard polymers such as PET and PAN is associated with the elevated PHI value, suggesting potential ecological risks and contributions from anthropogenic sources in Izmir Bay. The temporal dynamics of polymer occurrences are illustrated in the heatmap (Figure 8). The matrix reveals a strong year-round presence for PET and PE, which were detected in almost every sampling month. However, a pronounced seasonal increase is observed for PAN and rayon, particularly during the winter and early spring. The higher PHI values recorded in December and January coincide with increased representation of multiple high-hazard polymers. This pattern suggests that seasonal runoff and related hydrological processes may influence the diversity and distribution of polymers in Izmir Bay.

### 3.5. Relationship Between Fish Biometrics and MP Abundance

Statistical analyses were performed to determine whether fish size (total length and weight) or sex influenced the amount of microplastics ingested. According to the Spearman correlation test, there was no significant correlation between microplastic abundance and fish total length (r = 0.092, *p* = 0.331) or body weight (r = 0.084, *p* = 0.375).

**Figure 9:**
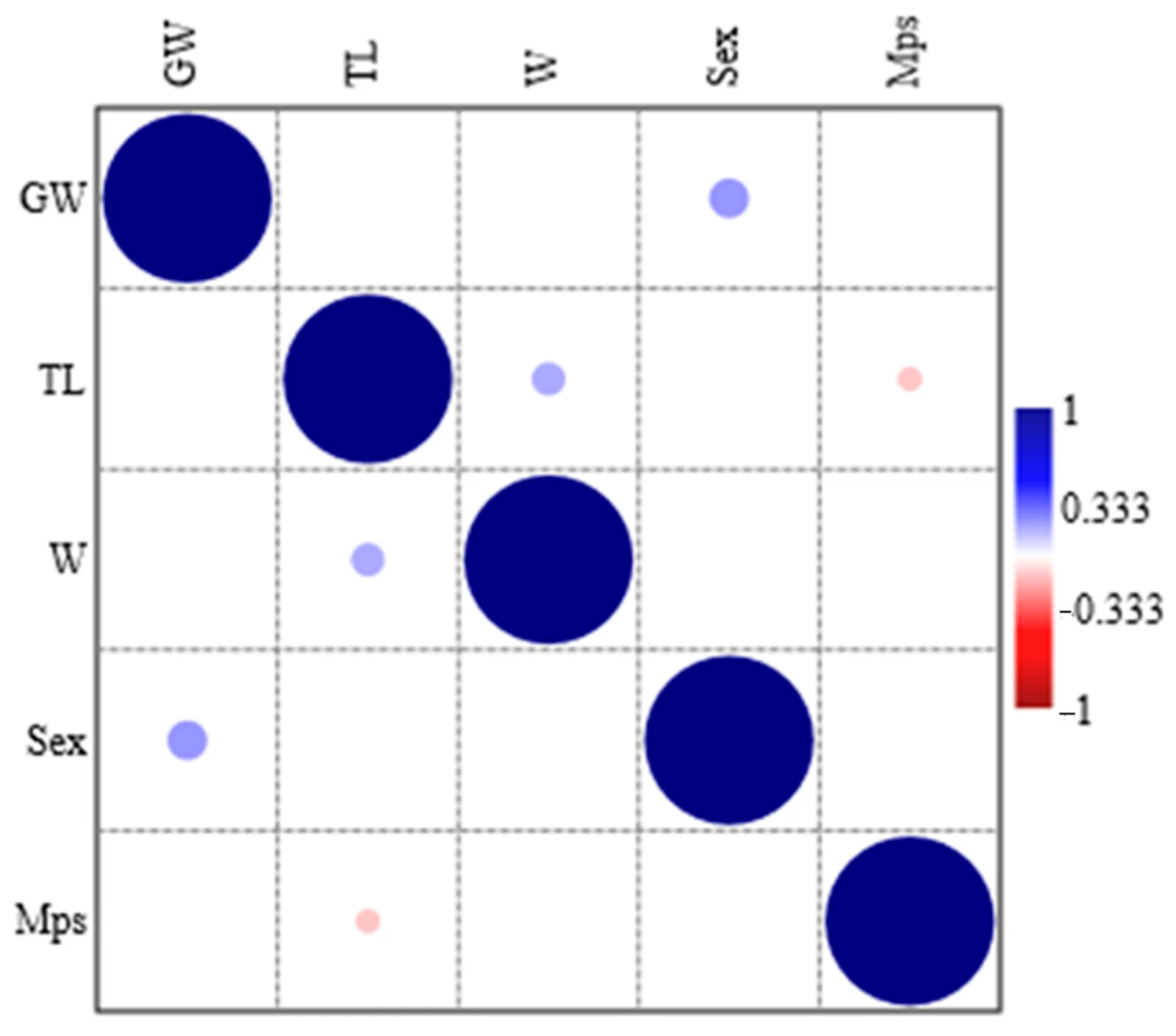
Relationship between fish biometrics and microplastic ingestion.

Similarly, no significant difference was observed in MP ingestion rates between male and female individuals (Kruskal–Wallis, *p* > 0.05). These results indicate that MP ingestion in *O. melanurus* is not size-selective or sex-dependent within the sampled population (Figure 9). This lack of correlation suggests a high and uniform bioavailability of microplastics in the water column of Izmir Bay, leading to equal exposure levels for all individuals across different life stages and sexes.

### 3.6. Hierarchical Cluster Analysis (HCA) of Monthly Variations

**Figure 10:**
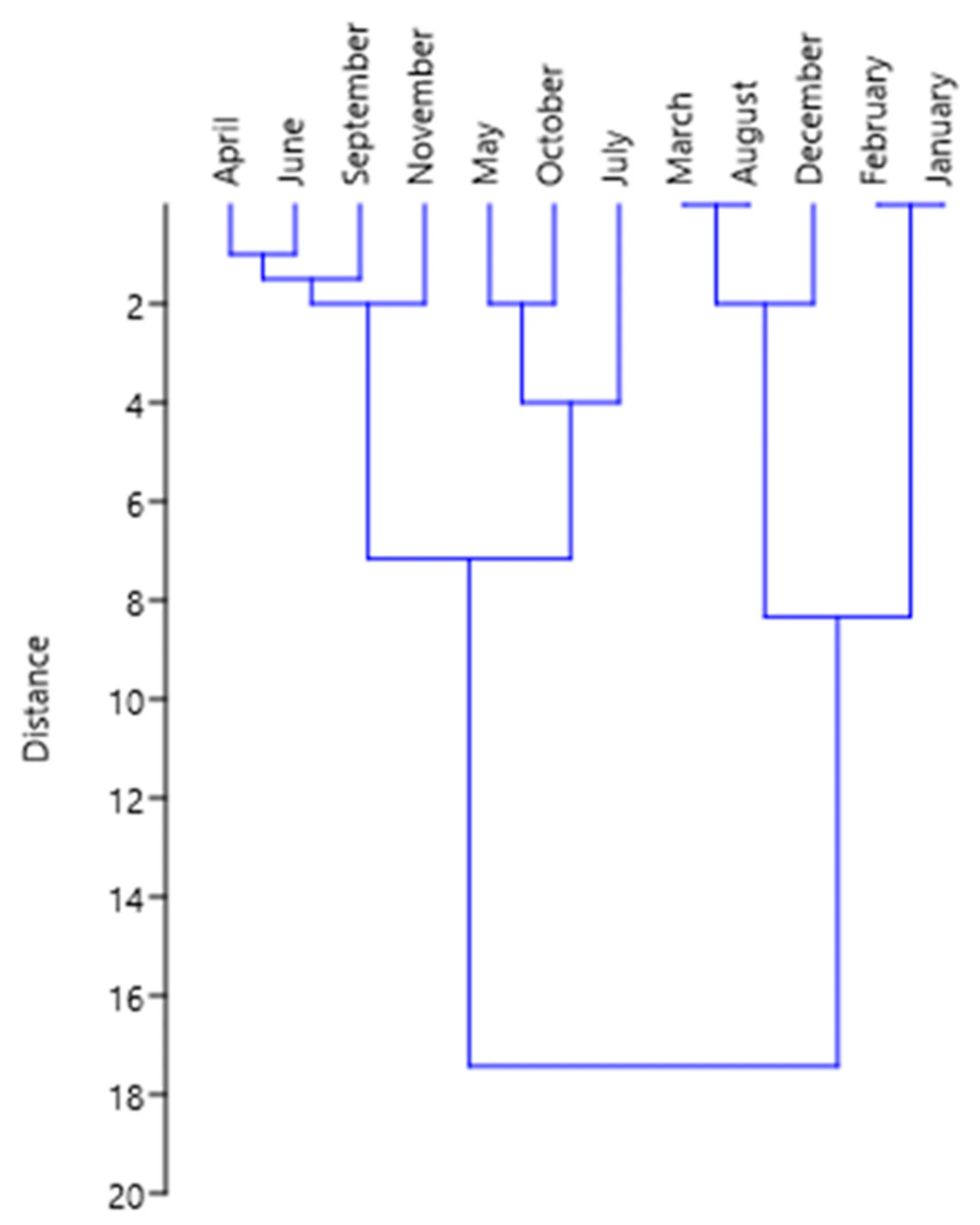
Dendrogram of Hierarchical Cluster Analysis (HCA).

A Hierarchical Cluster Analysis (HCA) was employed to statistically evaluate the temporal structuring of microplastic (MP) ingestion in *O. melanurus* (Figure 10). The resulting dendrogram exhibits a primary bifurcation at a prominent linkage distance (≈17.5), delineating two major seasonal clusters and confirming that MP pollution dynamics in Izmir Bay exhibit significant temporal heterogeneity rather than a uniform year-round pattern.

Cluster 1 (Baseline Ingestion Period): This broad cluster encompasses April, June, September, November, May, October, and July. The relatively low linkage distances within this primary group (ranging from ≈1.5 to 7.5) denote a period of comparative stability in MP bioavailability and ingestion rates. The tight sub-clustering of specific months (e.g., April–June and May–October at distances < 2.5) suggests similar environmental conditions and relatively consistent exposure levels during these periods, representing a baseline condition for the bay.

Cluster 2 (Elevated/Pulse Exposure Period): Diverging higher than from the baseline, this cluster comprises March, August, December, February, and January. The internal grouping of these months (merging at a linkage distance of ≈8.5) points to a distinct pollution profile. The aggregation of winter and early spring months (December through March) is consistent with increased land-based MP inputs driven by intense seasonal precipitation and urban runoff. Notably, the inclusion of late summer (August) within this precipitation-dominated group suggests the presence of secondary, non-meteorological drivers, potentially linked to peak maritime traffic, intensified coastal tourism, or specific late-summer hydrodynamic shifts in the bay.

The substantial linkage distance between these two temporal clusters demonstrates a fundamental shift in ecological risk (PHI) and exposure pathways, highlighting that specific seasonal events disproportionately affect MP ingestion levels.

## 4. Discussion

### 4.1. High Environmental Availability of Microplastics and Ingestion Patterns

This study demonstrates a substantial microplastic (MP) ingestion rate in *Oblada melanurus* from Izmir Bay, with an occurrence frequency of 63.9% and a mean abundance of 2.59 ± 0.41 items/individual. As detailed in Table 3, these values higher than exceed previously reported rates for this species across the Mediterranean basin, including adjacent Turkish coasts [38,65,66], the Adriatic Sea (25.4%) [67], and the Spanish Mediterranean (18.2%) [68]. Similarly, correlation analyses showed no statistically meaningful relationship between microplastic uptake and total length (TL), body weight (W), or gastrointestinal tract weight (GW). This absence of a strong biometric correlation suggests that microplastic ingestion in *O. melanurus* is likely driven by opportunistic feeding behavior and localized spatial-temporal exposure rather than individual body size or physiological capacity. While recent regional studies often identify polyethylene (PE) and polypropylene (PP) as the dominant polymers in commercial fish [38,69], our analysis reveals a distinct prevalence of polyethylene terephthalate (PET, 29%) and polyacrylonitrile (PAN, 14%). This divergence underscores the intense, localized impact of urban wastewater and textile discharges on the species foraging grounds, a phenomenon likely intensified by hydrodynamic entrapment within the bay’s semi-enclosed basin. Consistent with Kılıç et al. 2023, who evaluated polymer risk indices in the Northeastern Mediterranean, these findings indicate that habitat characteristics and specific anthropogenic inputs function as primary drivers of ecological risk [70].

The observation of a 100% occurrence frequency in December suggests that microplastic exposure may reach elevated levels during certain periods. The lack of a significant correlation between MP abundance and fish size (*p* = 0.331) indicates that environmental availability likely plays a more important role than size-selective feeding behavior. These findings suggest that microplastics are widely distributed in the water column, leading to non-selective ingestion across individuals, including different size classes. This interpretation is consistent with previous studies on Mediterranean fish species, which indicate that microplastic ingestion is largely influenced by local environmental contamination levels rather than species-specific biological or ecological traits [71,72].

*Oblada melanurus* may be especially vulnerable to the ingestion of fiber-type microplastics because it is an omnivorous benthopelagic species that feeds throughout the water column [38]. The predominance of black and blue microplastic particles (76% combined) may reflect the color distribution of particles in the surrounding environment. It could also be influenced by their visual resemblance to natural prey items. Similar observations have been reported for visually feeding fish, which may ingest microplastics resembling natural prey in terms of color, shape, and size [38]. The lack of a significant correlation between gastrointestinal tract weight (GW) and microplastic abundance suggests that MPs may also be ingested accidentally during normal feeding activities, a pathway that has been reported for omnivorous and filter-feeding fishes [38]. In addition, indirect trophic transfer through contaminated prey has been recognized as another potential route of MP uptake in marine organisms [38].

Hierarchical Clustering Analysis (HCA) identified two distinct periods of increased microplastic (MP) pollution, grouping the long winter-spring months (December–March) and August into a single high-impact cluster. These seasonal patterns may reflect different environmental processes. The winter-spring peak is thought to be associated with characteristic Mediterranean rainfall, consistent with the findings of Gündoğdu et al. (2017), who suggested that storm-induced runoff could be a significant source of MPs [43]. In İzmir, intense and prolonged rainfall extending into March could increase surface runoff and trigger Combined Sewer Overflows (CSO), bypassing wastewater treatment plant (WWTP) filtration and increasing the transport of textile-derived polymers (e.g., PET and PAN) into the gulf. This hypothesis is also consistent with recent assessments of the adjacent Gediz River, which reported distinct wet-season peaks in MP transport dominated by similar fiber and fragment morphotypes entering the Aegean coastal system [73]. In this context, future studies are needed that include relevant environmental variables and statistically evaluate the relationship between these variables and microplastic abundance.

**Table 3:** Comparison of MP ingestion in *O. melanurus* and other Sparidae species.

Region	Species	Occurrence (%)	Mean Abundance (Items/Ind)	Dominant Shape/Polymer	Reference
Izmir Bay (Aegean Sea)	*Oblada melanurus*	63.89	2.59 ± 0.41	Fiber/PET	Present Study
Izmir Bay	*Oblada melanurus*		5.14	Fiber/PP, PET	[38]
Izmir Bay	*Sparus aurata*		0.41 ± 0.1	Eva, PE, PMMA, PP, PVC	[69]
Mediterranean Sea	*Dentex gibbosus*	14	1		[74]
Mediterranean Sea	*Diplodus annularis*	42	2.85		[74]
Mediterranean Sea	*Pagrus pagrus*	78	1.20		[74]
Mediterranean Sea	*Pagellus acarne*	67	2.46		[74]
Mediterranean Sea	*Pagellus erythrinus*	52	1.21		[74]
Northeastern Mediterranean Sea	*Oblada melanurus*		0.9	Fiber, Pelet/PES, PE, PS	[66]
Mediterranean Sea, Spain, France, Italy and Greece	*Boops boops*	46.8	1.17 ± 0.07	Filaments, Fragment/PP, PE, PET, PVC, PS	[75]
Central Med. (Tunisia)	*Salpa salpa*		42.00 ± 6.08	Fiber/PP, PE	[76]
Portuguese Coast	*Diplodus vulgaris*	73	1.67 ± 0.27	Fiber/PA polyester, PP, rayon	[77]
Portuguese coast	*Boops boops*	60	0.80 ± 0.8	Fiber, Fragment/PP, PE, alkyd resin, rayon, polyester, nylon and acrylic	[78]

Note: Direct quantitative cross-study comparisons should be interpreted with caution due to methodological variations among published literature, including differences in lower size detection limits, digestion reagents, confirmation methods (e.g., visual vs. ATR-FTIR/Raman), sampled tissues (GI tract vs. whole organism), and reporting units.

The August peak, clustered with the wet season, represents a critical secondary driver. During late summer, Izmir Bay experiences reduced water circulation and elevated evaporation rates, leading to hydrodynamic entrapment conditions that promote the accumulation and concentration of MPs within the water column. This natural retention is further intensified by peak coastal tourism and increased maritime traffic, collectively enhancing local MP loading and producing pollution levels comparable to those observed during the rainy season.

The chemical makeup of Izmir’s urban pollution is clearly defined by a significant presence of black fibers (56%) and polymers commonly found in textiles, specifically PET (29%) and PAN (14%). While polyethylene (PE) and polypropylene (PP) are frequently reported as dominant in open-sea Mediterranean studies [79], the PET dominance in our samples highlights a strongly localized, land-based pollution source.

### 4.2. Toxicological Implications and Management Strategies

The calculated Polymer Hazard Index (PHI) of 129.52 places the detected polymer composition in Category IV (Danger), indicating a relatively high polymer hazard profile according to the PHI classification. Similar Category IV PHI values have also been reported for commercial marine fish species from adjacent Turkish waters, including the Marmara Sea [80]. This condition is likely exacerbated by the acidic environment of the GIT, which facilitates the leaching of adsorbed persistent organic pollutants (e.g., PCBs, PAHs) and endocrine-disrupting residual monomers from PET and PAN. Given the commercial prominence of this species, this bioaccumulation presents a concerning pathway for human exposure through the seafood supply chain.

To deal with these interconnected threats to the environment and public health, we need to make urgent changes to our infrastructure. For example, we need to add advanced tertiary treatment technologies (like membrane bioreactors, or MBR) to Izmir’s wastewater treatment plants to improve microfiber retention [81,82]. Concurrently, mitigating the seasonal winter–spring discharge pulse requires comprehensive CSO management through the separation of stormwater and domestic sewage networks to prevent untreated effluent bypasses [83]. In the end, *O. melanurus* is a reliable sentinel bioindicator species with great potential for application in long-term environmental monitoring of the Aegean Sea ecosystem due to the constant ingestion patterns and size-independent exposure seen in this study.

## 5. Conclusions

This study provides a comprehensive assessment of microplastic (MP) pollution in the gastrointestinal tracts of *Oblada melanurus* from Izmir Bay, revealing a critical ingestion frequency of 63.89%. The absence of a significant relationship between microplastic (MP) ingestion and fish size, together with the 100% occurrence rate observed in December, may indicate that MP exposure was widespread across the sampled individuals during this period. These temporal exposure patterns underscore the need for long-term monitoring to evaluate whether such extremes are becoming a persistent environmental condition rather than transient events. This confirms that municipal wastewater and textile-related effluents are the main causes of MP contamination, far more than general maritime litter. In order to better understand temporal variability and the durability of exposure patterns across time, long-term monitoring is necessary. This threshold denotes a situation in which microplastic (MP) exposure exerts a rather uniform ecological pressure over the entire population. The predominance of fibers (58%) and the high occurrence of PET (29%) and PAN (14%) suggest that these microplastics may originate from various anthropogenic sources, including plastic packaging, ropes, fishing gear, urban litter, and other plastic products. As highlighted by Kanwal et al. (2025), microplastics are released into the environment through multiple pathways, including waste mismanagement, road runoff, agricultural activities, wind abrasion of plastic materials, and the degradation of synthetic products [1]. Therefore, the presence of PET and PAN in the present study should be interpreted as being consistent with multiple potential sources of microplastic pollution rather than indicating a specific pollution source.

This study is one of the relatively few comprehensive analyses of the high-resolution temporal dynamics of microplastic (MP) ingestion, based on a rigorous monthly sampling regimen. The findings indicate an interaction between precipitation and human activity, as evidenced by Combined Sewer Overflow (CSO) events during winter and early spring (December–March) and by a hydrodynamic retention effect during the peak tourism period in August, when reduced water circulation coincides with increased anthropogenic pressure.

Furthermore, a PHI value of 129.52 places the bay in Category IV (Danger), indicating that chronic ingestion of such polymers may present substantial toxicological risks to *O. melanurus* and, as a result, may have implications for human health through the seafood chain. Management strategies to address these interlinked pressures may include a transition to advanced tertiary treatment technologies (e.g., membrane bioreactors) in regional wastewater treatment plants (WWTPs) and separation of stormwater and sewage networks. In general, the findings of this study suggest that *O. melanurus* has the potential to be a strong indicator species for assessing the success of future environmental policies and mitigation measures in the Aegean Sea ecosystem.

## Figures and Tables

**Table 1:** Taxonomic classification, biological characteristics, and microplastic (MP) ingestion metrics of *Oblada melanurus* collected from Izmir Bay (Eastern Aegean Sea, Türkiye).

Parameter	Value/Description
Taxonomic Information	
Family	Sparidae
Scientific name	*Oblada melanurus* (Linnaeus, 1758)
Common name	*Saddled seabream*
Ecological Data *	
Habitat	Benthopelagic
Diet	Omnivorous
Trophic Level	3.38
IUCN Conservation Status	Least Concern (LC)
Fisheries Data	
Average production (tons/2021–2022)	76.55
Biometric Measurements (*n* = 180)	
Total length (cm) (Mean ± SD)	22.71 ± 0.85
Body weight (g) (Mean ± SD)	141.61 ± 3.56
Gastrointestinal weight (g) (Mean ± SD)	2.80 ± 0.13
Microplastic Ingestion Metrics	
MP Occurrence (%)	63.89
Average MP abundance (items fish^−1^)	2.59 ± 0.41
Polymer Hazard Index (PHI)	129.52 (Category IV—Danger)

* The data were obtained from FishBase (Froese & Pauly, 2025) [45].

**Table 2:** Polymer types, relative abundances (*Pn*), assigned hazard scores (*Sn*), literature sources, and individual mathematical contributions to the overall Polymer Hazard Index (*PHI*) for microplastics detected in *Oblada melanurus*.

Material/Polymer Type	Relative Abundance (Pn, %)	Hazard Score (Sn)	Source of Hazard Score	Contribution to PHI (Pn × Sn)	Status in PHI Calculation
PET	35.0%	4	Lithner et al. (2011) [61]	1.40	Included
PE	25.0%	11	Lithner et al. (2011) [61]	2.75	Included
PP	20.0%	1	Lithner et al. (2011) [61]	0.20	Included
PA (Nylon)	10.0%	50	Lithner et al. (2011) [61]	5.00	Included
PVC	10.0%	10,511	Lithner et al. (2011) [61]	1051.10	Included
Calcite	—	N/A	Mineral Phase	N/A	Excluded
Rayon/Lyocell	—	N/A	Cellulosic Fiber	N/A	Excluded
OVERALL PHI	100%	—	—	1060.45	Category V (Extreme)

## Data Availability

The raw data supporting the conclusions of this article will be made available by the authors on request.
